# Friendly Home and Inhabitants' Morality: Mutual Relationships

**DOI:** 10.3389/fpsyg.2017.02348

**Published:** 2018-01-11

**Authors:** Sofya K. Nartova-Bochaver, Valeriya B. Kuznetsova

**Affiliations:** ^1^School of Psychology, National Research University “Higher School of Economics”, Moscow, Russia; ^2^Laboratory of Differential Psychophysiology of the Research Institute of Physiology and Basic Medicine, Novosibirsk, Russia

**Keywords:** personality, home environment, home environment functionality, home environment relevance, moral motive

## Abstract

The study is aimed at investigating the connection between the friendliness of the home environment and the moral motives' level. The friendliness of the home environment includes two aspects: the number of functions provided by home (functionality) and the congruence of these functions with inhabitants' needs (relevance). The theoretical framework of the study was formed by research and ideas emphasizing the interplay between people and their environments. We hypothesized that the friendliness of the home environment and inhabitants' moral motives would have a reciprocal relationship: the friendlier the home the higher the inhabitants' moral motives' level, and, vice versa, the higher the person's moral motives' level the more positive home image. The respondents were 550 students (25% male). The Home Environment Functionality Questionnaire, the Home Environment Relevance Questionnaire, and the Moral Motivation Model Scale were used. As expected, it was found that the friendliness of the home environment and the inhabitants' moral motives are in reciprocal synergetic relationships. Relevance formed more nuanced correlation patterns with moral motives than functionality did. Functionality predicted moral motives poorly whereas moral motives predicted functionality strongly. Finally, relevance and moral motives were found to be in mutual relationships whereas the perceived functionality was predicted by moral motives only.

I struggle with domestic entropy

As the source of divine energy

I conquer imperceptible blind forces

In an un-triumphant struggle

I wash dishes three times a day

I wash and polish the floor all over

I create meaning and structure in the world

On a spot that would seem to be empty

Dmitry Prigov

## Introduction

Seeking ecological ways to study personality and resources to stimulate people's well-being and personal growth is one of the most important trends in contemporary psychology (Schraube and Højholt, 2015). Everyday life practices and techniques are often considered to be the most reliable source of unguided self-therapy. Lang (1981, 1993) stated that psychology should not investigate artificial labor variables but rather people with their possessions in their rooms. As a strong eco-social resource, the home is used in the so-called apartment psychotherapy. In Jungian psychoanalysis, the home is a symbol of personality, reflecting its states and changes (Jung, 1964). It is difficult to overestimate the importance of the home environment as a regulating agent: it not only defines the circumstances of personal everyday life, but also is a “full member” of the family because of common experiences, history, and affordances to various activities provided by the home.

Recent studies showed that various aspects of people's relationships with their own homes were very favorable for their personalities. Xu et al. (2015) found that home attachment contributed to the self-efficacy, positive interpersonal relationships, as well as academic achievements, and prevented psychological disorders. It was also shown that a perceived positive home environment predicted well-being in mentally ill patients, namely, their psychiatric distress, recovery orientation, and adaptive functioning (Wright and Bret, 2007). Furthermore, the home environment decreased depression and self-alienation and increased self-esteem and resilience in health participants (Dmitrieva, 2014; Nartova-Bochaver S. et al., 2015; Nartova-Bochaver S. K. et al., 2015; Nartova-Bochaver et al., 2016). The home plays a significant role in the identity and meaning-of-life formation and helps adapting to a new life: “It has been argued that homes, artifacts, and objects not only express identity…but also help shape it” (Mazumdar and Mazumdar, 2009, p. 257). To sum up, the home contributes to inhabitants' normal functioning.

However, little is known about how the home is associated with “higher” personality abilities and strivings, such as morality, and what direction these associations could have—from the home to personality or backwards?

Hence, the aim of our research is to investigate how the home environment relates to inhabitants' moral motives.

## Theoretical framework

### Home and its inhabitants—why they resemble each other?

Husserl (1970) was one of the first philosophers who emphasized an inseparable connection between a person and the place where s/he lives. Human life is always “being-in-the-world,” and place (including home) contributes to the existence. Following him, Heidegger (1971) argued that “dwelling” is not only a routine activity that people perform at home but also is a way of existing in the world. It doesn't matter if the individual spends time at home or is away from it; the home image influences their personality. But how is this connection maintained and kept? The contemporary phenomenologists also believe that the origin of personality is rooted in nonverbal “being at home” experiences of childhood and inextricably associated with the place, space, and environmental objects (Korosec-Serfaty, 1985; Case, 1996).

After J. Uexkuell, every living thing is included in the so-called functional circle: being in the environment, it signifies firstly objects of the outside world according to its own needs (Merkwelt), and then, if necessary, appropriates or transforms them (Wirkwelt) (Kull, 2001). Relationships between the living thing and its environment are based on the feedback from the environment.

Similar phenomena are also described in human environments. Thus, Brunswik (1956) in his Lens Model emphasized a high person's selectivity in perceiving environmental objects which is led by person's preferences. According to Gibson (1986), every person selects affordances from an “objective” environment, satisfying his or her needs. Thus, inhabitants' representations of the home are always different and connected with their current states. Further, Gold (1980) in his behavioral geography also wrote about the circular connections between a person and environment. As a result, individuals and their environments acquire similar features, so that every individual gets her/his environmental identity.

Recent studies and theories also keep on the idea of mutual relations between a person and their environment. Thus, Person-environment Fit Theory examines the degree to which individual and environmental characteristics match (Edwards et al., 1998; Kahana et al., 2003). Theory of Behavioral Residues investigates everyday manifestations of personality due to which inhabitants personalize their homes (Gosling et al., 2002). Core affect explains how affects and appraisals or representations determine each other in the everyday life (Russell, 2003; Kuppens et al., 2012; Andringa and Lanser, 2013). We would also mention the Developmental Theory of Place Attachment which discovered processes underlying both attachment to place and attachment to people in this place (Morgan, 2010). This theory is of the highest value for the current study as it states that these attachments emerge in parallel with affected experiences of place in childhood. As grief, empathy, and pleasure develop and manifest in the specific place, social features, such as moral motives, are also expected to be connected with this place (in our case—home).

Last but not least we could also point out social anthropologists' works emphasizing mutual relationships between a person and their home; people perceive influences and change their environments at the same time (Ingold, 2002). They cannot conclude what happens earlier or later; but as a result, people get pertinent homes matching their personalities. Lang (1993) asked what the real research subject is: the house developing with the family or the family with the house. However, that may be, many studies state the inseparable connections between home and its inhabitants' personalities. Thus, the home determines inhabitants' “state of the soul,” and “state of the soul,” in turn, defines how inhabitants feel the world in general and their home as a part of the world.

### What does home mean in people's lives and how to assess it?

Scholars in anthropology, geography, sociology, and environmental psychology have emphasized a resurgence of interest in the home as a sort of “significant place” over the past four decades (Wright and Bret, 2007; Moser, 2009; Xu et al., 2015). They accentuate also the main cultural functions of the home, which are the following: defense, a place for communication and family, restoration, meditation, calm, privacy, silence, regulation one's moods, and core affect, and so on (Jung, 1964; Relph, 1976; Tuan, 1979; Harter, 1988; Russell, 2003; Kuppens et al., 2012; Andringa and Lanser, 2013). Home is an anti-pole of chaos (Bochaver, 2015), and, due to routine practices and everyday rules, it provides inhabitants with the feeling of order and life predictability. Moreover, home representations can put mental life of a person in order even s/he is away from home (Case, 1996). The very concept of “home” suggests that it is a key element in the development of people's sense of self (Hayward, 1975; Dovey, 1985; Altman and Low, 1992; Case, 1996; Gosling et al., 2002; Manzo, 2003; Easthope, 2004; Reznichenko, 2014; Nartova-Bochaver et al., 2016).

We define home as a unity of physical, social, and existential properties of a specific place satisfying inhabitants' needs. As a phenomenon, home is a multilevel substance. Thus, researchers identify such home representations as psychological, archetypical, everyday, ideal, and real homes (Manzo, 2003; Bochaver, 2015; Roster et al., 2016). All of them contribute to inhabitants' identities and personalities (Nartova-Bochaver et al., 2016). As home is much more than just a place, it could be considered as a palimpsest, including its own and inhabitants' history, changes in opinions, meanings, worldviews (Mitin, 2005; Barthes, 2015). In terms of post-modernism, it is a text which has been “written” by the all of inhabitant's experiences; that is why the home representation is expected to depend on their current opinions, including moral motives.

To assess home as a whole, we turned to the concept of environment friendliness (Harter, 1988; Greeno, 1994; Kyttä, 2004; Horelli, 2007; Coolen, 2011; Broberg et al., 2013). Friendliness in our study is not a feature of a family atmosphere but rather a positive metaphoric description of the comfortable and convenient home as a physical environment with its spaces, facilities, and things. Initially, this term was developed in information science (“user-friendly” or “human-friendly” interface). As every environment, by and large, is “interface” between a person and the world, this term has become popular, especially in the area of child environments research. Thus, Kyttä (2004) considers child environment friendly if it provides a variety of resources, and these resources are available to a child. We regard as friendly that home which includes plenty of functions (functionality) most of which are necessary for inhabitants, have been used by them, and attract positive emotions (relevance). Functionality includes different affordances: thus, it could be, for example, an opportunity to use the Internet, to keep privacy, to plan one's own daily schedule, to arrange a party, etc. In fact, it is an objective description of the home environment that could be used for expertise purpose but it cannot show whether a real inhabitant needs it. For example, there is the Internet at home (which is good, of course) but a person doesn't need and doesn't use it. However, if there are many functions at home, it is easier, to choose among them the most important ones for a person.

The relevance of the home environment shows how many of the home functions are wanted by inhabitants and available to their usage. Thus, it could happen that the convenient and comfortable home is disliked by the person for any (sometimes irrational) reasons and doesn't fit her/him. When we talk about the relevance, we consider home as a holistic structure matching the dwellers, influencing them and being influenced by them at the same time. In other words, the relevance reflects “compatibility” between people and their home. Comparing functionality and relevance, in the first case, we get a more or less “objective” picture of home; in the second one—information on how comfortable inhabitants feel and how pertinent homes are to them.

Relevance depends on functionality; if there are few functions at home it is difficult to choose from them the most wanted. But this dependence is not full: it could happen that there are many functions but no one of them matches the person. Moreover, the previous studies showed that influences of functionality and relevance on personality were different (Nartova-Bochaver et al., 2016).

### Moral motives as a form of personal and social regulation

The second variable measured in our study was inhabitants' moral motives. Morality is very closely connected to the rules introduced and accepted by people. Just as home puts into order the inhabitants' living reality, giving them implicit and explicit rules, morality regulates the individual's mental world and provides people with permissions and prohibitions in their lives.

During the last decades, a new trend appeared in the area of the moral research which (in contrast to earlier understanding of morality as a special spiritual gift) is close to the pragmatic interpretation of good or bad actions in everyday life. From this perspective, morality is considered not a target personality characteristic but a predictor of the adaptive and successful behavior (Fredrickson, 2004; Haidt and Joseph, 2004; Wood et al., 2010; Wenzel and Okimoto, 2012; Janoff-Bulman and Carnes, 2013). Good motives and personal features, such as love, justice, gratitude, and forgiveness, are investigated concerning their roles, as factors or triggers, in the acquisition of the life success. However, non-direct their influence might be, it increases the probability of a favorable feedback from the society and environment. People who were initially moral, broaden their favorable world view, became more optimistic and less stressful, and, as a result, arranged a positive image of life (Fredrickson, 2004).

Like environmental behavior, morality is often beyond rationalization. The Moral Foundations Theory suggests that moral judgments are caused by quick moral intuitions but not reasoning (Haidt and Joseph, 2004), with the prior role playing by unconscious moral features—attitudes and motives. According to this theory, individuals possess four “intuitive ethics,” which stem from the human evolution as responses to adaptive challenges. These four ethics—suffering, hierarchy, reciprocity, and purity are, first, tightly connected with culture, and, secondly, strongly predict individuals' world view, especially political and religious attitudes (Balzer, 2012).

In turn, Wenzel and Okimoto (2012) argue that ability to forgive provides individuals with benefits of psychological well-being, and Wood et al. (2010) demonstrated what people profited from their gratitude trait.

In our research, we turned to one of the most popular models, the Moral Motivation Model by Janoff-Bulman, which is based on the fundamental psychological distinction in the motivation and self-regulation of behavior—approach vs. avoidance, or behavioral activation versus inhibition (Carver, 2006; Janoff-Bulman and Carnes, 2013). This dual regulatory system is recognized across multiple areas of psychology.

According to Janoff-Bulman's theory, we have to distinguish between proscriptive and prescriptive morality. The first, based on avoidance, shows the person what he or she should not do; it involves restraining a “bad” motivation and helps in overcoming temptations or impulsive actions. To sum up, morality of this sort protects from harm. Prescriptive morality, based on approach, focuses on what he or she should do; it stimulates doing something good. Most generally, prescriptive morality awakes helping, sharing motives, and so on. Thus, we have two aspects of moral regulation. In addition, Janoff-Bulman identified three kinds of social targets morality is directed toward: self, other, and a group. In total, there are six moral motives. Proscriptive motives are as follows: self-restraint (self), not harming (other), social order (group). Prescriptive motives are self-reliance, or industriousness (self), helping/fairness (other), and social justice (group).

Although some ideas of Janoff-Bulman's theory are considered to be a point for discussion, this model is in accordance with another respectable approach to investigation into “higher” personal features, namely, the Schwartz Value Theory (Fontaine et al., 2008). Thus, we could draw a parallel line between moral motives and more stable features, values, such as self-restraint—self-discipline, not harming—humble, self-reliance—ambitious and competent. Both theories include social order, social justice, and helping, as motive and values. Moreover, both of them emphasize the fact that motive and values can stabilize or stimulate a person, and define orientation of his/her efforts. Finally, what is more important, World values survey conception integrates moral values with values of sense of belonging, national and family security, in other words, place and home.

### Are inhabitants of a friendly home more moral, or do moral people perceive their homes as friendlier ones?

The short previous review has shown that the home is a basic environment which realizes a variety of functions in human lives. As Case (1996) found, home can be used dialectically; two conditions are affected: (1) routine—a break from routine, and (2) being with other people—being alone. To sum up, the home both stabilizes and stimulates its inhabitants in various activities. In other words, the home is a source of conscious or unconscious rules, regulating people's adaptation and growth.

As shown earlier, the home environment predicts inhabitants' mental health (Nartova-Bochaver S. et al., 2015; Nartova-Bochaver S. K. et al., 2015). But little is known about the possible inter-influence of home and people's higher features, namely, morality. Why do we expect to reveal any connections between the home environment and dwellers' moral motives?

First, we can refer to the idea of “proprium” by Allport (1961) who described a consequence of personality maturing with moral values growing after and from body and territorial aspects of the personality. In line with Allport's study stood the work by Maslow, who stated that “higher” needs appear after the basic needs for safety, stability, and so on have been satisfied (Maslow et al., 1970). Thus, if people feel they are living in convenient, comfortable circumstances, they are more likely to practice a moral worldview as compared to those who are living in adverse situations.

Second, studies on moral development have discovered that moral representations and attitudes depend not only on personality but also on the situation or environment (Oser, 1986; Nartova-Bochaver, 1993; Schmitt et al., 2009; Heinrichs et al., 2013). Despite the lack of the direct evidence for the role of home in this influence, people in friendly homes are expected to practice a trusting worldview. As they feel safer and do not spend energy on defending themselves from chaos or troubles, they could become kinder and more moral personalities.

Further evidence for our expectations is given by environmental psychologists and moral geographers who emphasized a tight connection between place attachment and judgments on justice (however, they did not consider the home as a place) (Zajonc, 1968; Smith, 2000; Clayton and Opotow, 2003). Finally, the home is also a place where people can regulate their moods and core affect, and, as a result, develop their attitudes toward other people (Russell, 2003; Kuppens et al., 2012).

At the same time, morality as a factor of person's functioning has not been attracting researchers' attention until now; much more often morality, being an important social value, is investigated as a dependent variable. Although evidence for considering moral motives a predictor of the home representation is scant, we could refer to the results from Moral Foundations Theory and positive psychology. Thus, it was shown that specificity of ethics contributed to whether a person endorsed liberal or conservative opinions, which, in turn, defined a so-called person's “implicit political identity” and other behavioral consequences (Graham et al., 2009). As home is a significant part of fatherland, there are expected connections between moral motives' level and the image of home. Every policy starts from attitude to the home as a value. In line with these results, Balzer (2012) has revealed that not only political but also religious attitudes depend on the ethics. As home is a specific object in everyday life it could be sometimes sacred, sometimes perceived rationally but in any case a part of a world representation. Further evidence for assuming that moral motives might predict home representation was taken from the Broaden-and-build theory of positive emotions (Fredrickson, 2004). She stated that positive emotions contributed to the perception of reality as more friendly and just. As love, being a base of all moral motives, concerns individual's relatives who share with him/her the home environment, in the first place, it is expected to concern home as well. According to Morgan (2010), attachment to people is tightly connected with attachment to the place which these people inhabit.

To sum up, as both our variables, feeling of friendly home, and moral motives, belong to the area of the person's worldview, participate in the person's interaction with environments, and shape rules determining these participations, we could predict them to be inter-connected. In addition, we expect this inter-connection to be reciprocal: people who are living in the friendly home feel safe = > they are friendly to the world and to people = > they receive from the world and people positive feedback = > they like their home (as a part of the world) even greater, etc.

Because of the lack of previous outputs in this field, we planned an exploratory investigation aiming at discovering any new links between home environment and personal moral motives. We hypothesized that friendliness of the home environment and inhabitants' moral motives would be in reciprocal relationships: (1) the friendlier the home the higher inhabitants' moral motives' level, and, vice versa, (2) the higher person's moral motives' level the more positive home image.

## Materials and methods

### Procedure and sample

The sample consisted of 550 respondents (24% male; 35 respondents did not indicate their gender) aged 17–30 (*M* = 20.6, *SD* = 2.3). Some of them failed to complete the survey and were excluded from the analysis. Data were predominantly collected in Moscow universities. All participants of the study signed the form of informed consent; data was collected in class as a part of individual home work in Psychology of individual differences. Most respondents (92%) filled in the questionnaires on-line or using a “pencil-and-paper” procedure in the university room; some respondents (8%) did from home. Participation was voluntary and evaluated as an elective (extra) part of credit in this subject.

The study was approved by the National Research University Higher School of Economics Committee on Interuniversity Surveys and Ethical Assessment of Empirical Research.

### Measurement instruments

To measure the friendliness of the home environment, we developed a tool set consisting of two scales (the Functionality of the Home Environment Questionnaire [FHEQ] and the Relevance of the Home Environment Questionnaire [RHEQ]; see Table 1). These questionnaires included constructs associated with specific affordances of the home environment or inhabitants' needs (Nartova-Bochaver S. K. et al., 2015; Nartova-Bochaver et al., 2016).

**Table 1:** Measures and their description.

**No**.	**Scale**	**Content**	
1	Pragmatism 25 items	Description of those simple everyday functions without which the home becomes inconvenient	
2	Development 12 items	Properties of the home environment that stimulate personal development (e.g., supply sensory, cognitive, social information, or maintaining the inhabitant's identity)	
3	Stability 7 items	Psychological and physical stability and predictability necessary for home recognition as a living space	
4	Protection 11 items	Self-presentation, presentation of the resident's status and power, and aesthetic needs	
**THE RELEVANCE OF THE HOME ENVIRONMENT QUESTIONNAIRE**			
1	Management of the home environment 27 items	The capability to control and predict the home environment context	
2	Potential 19 items	Constructs associated with home supporting and stability	
3	Self-presentation 17 items	The inhabitants' possibility to personalize their own space and to signify the individual and social characteristics of the dwellers through the home environment	
4	Ergonomics 17 items	Home environment convenience and aesthetics	
5	Home detachment 12 items	Reasons of home estrangement, loss of home attachment, and sense of belonging; discomfort, inconvenience, low functionality of a living space, and lack of desire to come back home	
6	Plasticity 9 items	The capability of the home environment to be dynamic in accordance with the changing resident's needs	
7	Historicity 7 items	The links of the home with personal, family, and general pasts	
**THE MORAL MOTIVATION MODEL SCALE**			
1	Self-restraint 5 items	Inhibition (proscriptive motives)	Self (personal)
2	Not harming 5 items		Other (interpersonal)
3	Social order 5 items		Group (collective)
4	Self-reliance (industriousness) 4 items	Activation (prescriptive motives)	Self (personal)
5	Helping/fairness 5 items		Other (interpersonal)
6	Social justice 5 items		Group (collective)

The FHEQ consists of 55 statements; responses on these scales were made on a seven-point scale ranging from 1 (*my home cannot …at all*) to 7 (*my home can …very much*) to indicate the extent to which participants agreed with the statements. It has a four-factor structure, supported in a previous study (Nartova-Bochaver et al., 2016), and it discloses affordances provided by the home. Cronbach's α was from 0.69 to 0.88 with a mean of 0.79.

Examples of FHEQ items are these: “My home can…demonstrate the level of wealth, be accessible (geographically and financially), be spacious, give an opportunity to care for myself, give an opportunity to eat at my own pace, give an opportunity to sleep when I want to, be a shelter,” and so on.

The RHEQ contains 108 items and measures how much individual needs are satisfied by the home. Responses on these scales were made on a five-point scale ranging from 1 (*does not apply to me at all*) to 5 (*fully applies to me*). The questionnaire includes seven scales (Nartova-Bochaver S. et al., 2015). Cronbach's α was from 0.75 to 0.89 with a mean of 0.82.

Examples of RHEQ items are these: “I ‘feel at home’ in my home,” “People at my home have a good job and income,” “I have no need to buy my own house,” “My house can ‘talk’ to a guest about my victories and hobbies,” “Looking at my room, it is hard to say how old I am,” “Whatever happens (even after a quarrel with relatives) I know I can come back home.”

To measure the moral motives level, we used the Moral Motivation Model Scale, which consists of six scales and 29 items (see Table 1). The scale reflects the moral motives identified by Janoff-Bulman (Janoff-Bulman and Carnes, 2013). Responses on these scales were made on a seven-point scale ranging from 1 (*strongly disagree*) to 7 (*strongly agree*). Cronbach's α was from 0.63 to 0.79 with a mean of 0.71.

Examples of the MMM scale items are these: “It's particularly important to me to demonstrate self-control in the face of temptation,” “I think it's important to take responsibility for my failures and setbacks rather than blame other people,” “Being generous is an important part of who I am,” “Giving to groups worse off in society does not make those groups too dependent on help.”

Statistical analysis was performed using SPSS 22.

## Results

### Connections between the home environment and moral motives

As expected, many positive connections (in total, 42 links) between moral motives and features of the friendly home were found, with the exception of home detachment which was connected with self-reliance negatively (see Table 2). This fact is in accordance to earlier received data about the destroying function of the home detachment in all of the investigated personal states and activities (Nartova-Bochaver et al., 2016; Roster et al., 2016). The most nuanced correlation patterns were formed by self-reliance with relevance, namely, management (*r* = 0.26, *p* = 0.000), potential (*r* = 0.32, *p* = 0.000), plasticity (*r* = 0.16, *p* = 0.000), self-presentation (*r* = 0.21, *p* = 0.000), ergonomics (*r* = 0.27, *p* = 0.000), and negative with home detachment (*r* = −0.22, *p* = 0.000). Relations between self-reliance and all parameters of functionality were also very strong. There were found correlations with pragmatism (*r* = 0.22, *p* = 0.000), development (*r* = 0.17, *p* = 0.000), stability (*r* = 0.15, *p* = 0.001), and with protection (*r* = 0.17, *p* = 0.000). Social order motive formed links with all of relevance features, except home detachment: management (*r* = 0.10, *p* = 0.043), potential (*r* = 0.21, *p* = 0.000), plasticity (*r* = 0.16, *p* = 0.000), self-presentation (*r* = 0.10, *p* = 0.026), ergonomics (*r* = 0.19, *p* = 0.000) and, finally, historicity (*r* = 0.10, *p* = 0.024). Moreover, we found strong positive correlations between social order and all of functionality features—pragmatism (*r* = 0.14, *p* = 0.002), development (*r* = 0.14, *p* = 0.002), stability (*r* = 0.15, *p* = 0.001), and protection (*r* = 0.15, *p* = 0.001). Helping/fairness motive was found to be connected with five out of seven features of relevance: management (*r* = 0.12, *p* = 0.009), potential (*r* = 0.21, *p* = 0.009), self-presentation (*r* = 0.12, *p* = 0.007), ergonomics (*r* = 0.18, *p* = 0.000), and historicity (*r* = 0.15, *p* = 0.001). Its connections with functionality were also significant—with pragmatism (*r* = 0.14, *p* = 0.001), development (*r* = 0.15, *p* = 0.001), stability (*r* = 0.11, *p* = 0.018), and protection (*r* = 0.12, *p* = 0.009). Only self-restraint motive differed from all listed above as it was linked positively with two features of friendly home—potential (*r* = 0.10, *p* = 0.041) and ergonomics (*r* = 0.10, *p* = 0.033).

**Table 2:** Correlations of the moral motives and the home environment features.

**Measure**	**Helping/fairness**	**Not harming**	**Social justice**	**Social order**	**Self- reliance**	**Self- restraint**
	**Other**	**Group**	**Self**			
**THE RELEVANCE OF THE HOME ENVIRONMENT**						
Management	0.12, 0.009	0.11, 0.013	ns	0.10, 0.043	0.26, 0.000	ns
Potential	0.21, 0.009	0.19, 0.000	0.16, 0.000	0.21, 0.000	0.32, 0.000	0.10, 0.041
Home detachment	ns	ns	ns	ns	−0.23, 0.000	ns
Plasticity	ns	0.10, 0.032	0.13, 0.003	0.14, 0.002	0.16, 0.000	ns
Self-presentation	0.12, 0.007	0.14, 0.002	0.13, 0.005	0.10, 0.026	0.21, 0.000	ns
Ergonomics	0.18, 0.000	0.22, 0.000	0.21, 0.000	0.19, 0.000	0.27, 0.000	0.10, 0.033
Historicity	0.15, 0.001	0.12, 0.007	0.12, 0.000	0.10, 0.024	ns	ns
**THE FUNCTIONALITY OF THE HOME ENVIRONMENT**						
Pragmatism	0.14, 0.001	ns	ns	0.14, 0.002	0.22, 0.000	ns
Development	0.15, 0.001	ns	ns	0.14, 0.002	0.17, 0.000	ns
Stability	0.11, 0.018	ns	ns	0.15, 0.001	0.15, 0.001	ns
Protection	0.12, 0.009	ns	ns	0.15, 0.001	0.17, 0.000	ns

*First number is a correlation index; second number is a level of significance. Prescriptive motives are in gray*.

It seems also to be interesting that, whereas a half of motives (helping/fairness, social order, and self-reliance) were connected with both functionality and relevance, the rest of them (not harming, social justice, and self-restraint) formed links only with the relevance scores. Thus, not harming and social justice were strongly connected with potential (respectively, *r* = 0.10, *p* = 0.000; *r* = 0.16, *p* = 0.000), plasticity (respectively, *r* = 0.10, *p* = 0.032; *r* = 0.13, *p* = 0.003), self-presentation (*r* = 0.14, *p* = 0.002; *r* = 0.13, *p* = 0.005), ergonomics (*r* = 0.22, *p* = 0.000; *r* = 0.21, *p* = 0.000), and historicity (*r* = 0.12, *p* = 0.007; *r* = 0.12, *p* = 0.000). In addition, not harming was connected with management (*r* = 0.11, *p* = 0.013) and plasticity (*r* = 0.10, *p* = 0.032).

Then, we performed a hierarchical regression analysis to determine whether the home environment adds a unique variance into moral motives and vice versa, whether moral attitudes could predict home environment images.

### Home environment as a predictor of moral motives

First of all, we found that age and gender did not predict moral motives; thus, we could conclude that this connection is not age or gender sensitive, at least in our mainly youth sample (Tables 3, 4). Surprisingly, we have revealed than functionality of the home environment did not play a significant role as a predictor of the moral motives. Only self-reliance was predicted by plasticity (β = 0.24, *p* = 0.007, f^2^ = 0.05) which means that people who are living in the changeable homes tend to be industrious and rely on themselves.

**Table 3:** Predictive validity of the functionality of the home environment for the moral motives.

**Dependent variable**	**Step**	***R***	***R*^2^**	**Predictor**	**f^2^**	**β**	***p***	***F***	***p***	**df**
Self-reliance	1	0.08	0.01	Gender		0.06		1.5		455
				Age		0.05				
	2	0.23	0.05	Pragmatism	0.05	0.24	0.007	4.4	0.000	457

**Table 4:** Predictive validity of the relevance of the home environment for the moral motives.

**Dependent variable**	**Step**	***R***	***R*^2^**	**Predictor**	**f^2^**	**β**	***p***	***F***	***p***	**df**
Helping/fairness	1	0.07	0.001	Gender		0.07		1.2		438
				Age		0.01				
	2	0.06	0.04	Potential	0.03	0.25	0.006	2.9	0.02	331
				Historicity	0.01	0.11	0.04			
Not harming	1	0.08	0.002	Gender		0.08		1.4		438
				Age		0.02				
	2	0.24	0.04	Ergonomics	0.20	0.22	0.006	2.9	0.002	431
Social justice	1	0.02	0.01	Gender		0.02		0.12		438
				Age		−0.00				
	2	0.24	0.06	Home detachment	0.15	0.13	0.032	2.9	0.002	431
				Ergonomics	0.22	0.23	0.005			
Social order	1	0.08	0.01	Gender		0.07		1.5		438
				Age		0.05				
	2	0.31	0.10	Management	0.01	−0.18	0.029	5.2	0.000	431
				Potential	1.33	0.34	0.000			
				Self-presentation	0.04	−0.18	0.024			
				Ergonomics	0.01	0.17	0.035			
Self-reliance	1	0.10	0.01	Gender		0.09		2.04		438
				Age		0.04				
	2	0.37	0.14	Potential	0.41	0.26	0.003	7.5	0.000	431
				Home detachment	0.14	−0.12	0.028			

*Prescriptive motives are in gray*.

Furthermore, we found that five out of six motives are significantly predicted by the home relevance (see Table 4). Thus, prescriptive, activation motives are predicted: helping by historicity and potential (respectively, β = 0.11, *p* = 0.040, f^2^ = 0.01; β = 0.25, *p* = 0.006, f^2^ = 0.03), social justice by ergonomics (β = 0.23, *p* = 0.005, f^2^ = 0.22), and self-reliance by potential (β = 0.26, *p* = 0.003, f^2^ = 0.41). Interestingly, home detachment predicted social justice (β = 0.13, *p* = 0.032, f^2^ = 0.15). As for prospective motive not harming and social order, they are predicted by ergonomics (respectively, β = 0.22, *p* = 0.006, f^2^ = 0.20; β = 0.17, p = 0.035, f^2^ = 0.01); in addition, social order is predicted by potential (β = 0.34, *p* = 0.000, f^2^ = 1.33). The more ergonomic the home is the more likely its inhabitants are ready to take into consideration other people. Finally, plasticity did not predict any moral motives at all. To sum up, the results are not simple: contrary to our expectations, self-presentation (β = −0.18, *p* = 0.024, f^2^ = 0.04) and management (β = −0.18, *p* = 0.024, f^2^ = 0.01) turned out to be anti-predictors of social order. In total, we have found 11 connections home environment = > moral motives.

### Moral motives as predictors of the home environment representations

We have also been exploring how moral motives predicted perceived home environment features, and we have got very impressive results. According to the expectations, people who have high moral motives level tend to perceive their homes as more functional, e.g., more pragmatic, giving opportunities for development, stable, and protective (see Table 5). More specifically, social order motive made impact on all parameters of the functionality—pragmatism (β = 0.12, *p* = 0.030, f^2^ = 0.09), development (β = 0.11, *p* = 0.048, f^2^ = 0.08), stability (β = 0.15, *p* = 0.005, f^2^ = 0.01), and protection (β = 0.15, *p* = 0.007, f^2^ = 0.01). Self-reliance contributed to development (β = 0.14, *p* = 0.005, f^2^ = 0.02), stability (β = 0.14, *p* = 0.004, f^2^ = 0.02), and protection (β = 0.15, *p* = 0.003, f^2^ = 0.02), helping motive—to development (β = 0.14, *p* = 0.030, f^2^ = 0.28). Social justice, not-harming, and self-restraint did not predict perceived functionality of the home.

**Table 5:** Predictive validity of the moral motives for the perceived functionality of the home environment.

**Dependent variable**	**Step**	***R***	***R*^2^**	**Predictor**	**f^2^**	**β**	***p***	***F***	***p***	**df**
Pragmatism	1	0.04	0.001	Gender		0.02		0.29		472
				Age		0.03				
	2	0.26	0.07	Social order	0.09	0.12	0.030	4.3	0.000	474
Development	1	0.03	0.001	Gender		0.01		0.23		479
				Age		0.03				
	2	0.23	0.05	Helping/fairness	0.28	0.14	0.030	3.2	0.002	473
				Social order	0.08	0.11	0.048			
				Self-reliance	0.02	0.14	0.005			
Stability	1	0.03	0.01	Gender		0.02		0.24		479
				Age		0.02				
	2	0.22	0.05	Social order	0.01	0.15	0.005	3.1	0.002	473
				Self-reliance	0.02	0.14	0.004			
Protection	1	0.04	0.01	Gender		0.01		0.31		481
				Age		−0.04				
	2	0.22	0.05	Social order	0.01	0.15	0.007	3.1	0.002	475
				Self-reliance	0.02	0.15	0.003			

*Prescriptive motives are in gray*.

Furthermore, we analyzed predictive validity of the moral motives for the relevance of the home environment (see Table 6). We found that, in accordance to our assumptions, six out of seven parameters of the relevance were predicted by inhabitants' moral motives. Self-reliance made significant impact on management (β = 0.29, *p* = 0.000, f^2^ = 0.06), potential (β = 0.29, *p* = 0.000, f^2^ = 0.08), plasticity (β = 0.16, *p* = 0.001, f^2^ = 0.02), self-presentation (β = 0.21, *p* = 0.000, f^2^ = 0.03), and ergonomics (β = 0.24, *p* = 0.000, f^2^ = 0.05). Social order contributed to potential (β = 0.13, *p* = 0.012, f^2^ = 0.01) and plasticity (β = 0.11, *p* = 0.041, f^2^ = 0.01). Not-harming predicted ergonomics (β = 0.13, *p* = 0.043, f^2^ = 0.02). In addition, self-reliance was anti-predictor of the home detachment (β = −0.28, *p* = 0.000, f^2^ = 0.05): the more industrious the person is the more seldom he/she meets situations of domestic stress. Contrary to our expectations, self-restraint was anti-predictor of the following features of the perceived friendly home: management (β = −0.12, *p* = 0.025, f^2^ = 0.01), potential (β = −0.11, *p* = 0.036, f^2^ = 0.01) and ergonomics (β = −0.11, *p* = 0.036, f^2^ = 0.01). At the same time, it contributed to the home detachment (β = 0.11, *p* < 0.05). In addition, it was shown that helping motive impacted the perceived plasticity of the home negatively (β = −0.15, *p* = 0.024, f^2^ = 0.003), and historicity was not predicted by moral motives at all. In total, we have found 23 connections moral motives = > home environment.

**Table 6:** Predictive validity of the moral motives for the perceived relevance of the home environment.

**Dependent variable**	**Step**	***R***	***R*^2^**	**Predictor**	**f^2^**	**β**	***p***	***F***	***p***	**df**
Management	1	0.03	0.001	Gender		0.03		0.18		472
				Age		0.01				
	2	0.29	0.09	Self-restraint	0.01	−0.12	0.025	5.4	0.000	466
				Self-reliance	0.06	0.29	0.000			
Potential	1	0.03	0.001	Gender		0.02		0.27		480
				Age		−0.02				
	2	0.37	0.14	Social order	0.01	0.13	0.012	9.3	0.000	474
				Self-restraint	0.01	−0.11	0.036			
				Self-reliance	0.08	0.29	0.000			
Home detachment	1	0.07	0.01	Gender		−0.05		1.22		488
				Age		0.06				
	2	0.29	0.08	Self-restraint	0.01	0.11	0.029	5.3	0.000	482
				Self-reliance	0.05	−0.28	0.000			
Plasticity	1	0.02	0.00	Gender		−0.01		0.07		490
				Age		0.01				
	2	0.24	0.06	Helping /fairness	0.003	−0.15	0.024	3.6	0.000	484
				Social justice	0.01	0.13	0.037			
				Social order	0.01	0.11	0.041			
				Self-reliance	0.02	0.16	0.001			
Self-presentation	1	0.07	0.01	Gender		0.06		1.2		482
				Age		−0.04				
	2	0.25	0.05	Self-reliance	0.03	0.21	0.000	4.1	0.000	476
Ergonomics	1	0.04	0.01	Gender		0.03		0.44		482
				Age		−0.03				
	2	0.34	0.11	Not harming	0.02	0.13	0.043	7.6	0.000	476
				Social justice	0.01	0.12	0.039			
				Self-restraint	0.01	−0.11	0.036			
				Self-reliance	0.05	0.24	0.000			

*Prescriptive motives are in gray*.

Therefore, our study revealed the predictive role of the friendliness of the home environment in the moral motives as well as the predictive role of the moral motives in the friendly home representation.

## Discussion

Our study demonstrated that, as expected, home and its inhabitants were in the mutual synergetic relationship: in a friendly home, people demonstrated higher morality, and, vice versa, moral people generated positive attitudes toward both other people and home as a place where people live. In other words, it showed that the inner and the outside worlds are maintained in parallel, and are in fact influenced by each other. These results are in line with Husserl's understanding of integrity of the person and their being (Husserl, 1970) as well as with Uexkuell's idea of the “functional circle” including cycles of person-environment interactions (Kull, 2001). Moreover, they could be interpreted based on the Theory of Lens by Brunswik (1956) and the Developmental Theory of Place Attachment by Morgan (2010).

We also revealed that both parameters of a friendly home, functionality, and relevance, were positively connected with the moral motives but relevance formed more connections. Indeed, whereas functionality concludes characteristics of the “objective,” de-personalized comfort, relevance takes into account personality of inhabitants—his/her needs and preferences. Possibly, that is why just relevance, unlike functionality, can awake “higher” moral motives in people. Moreover, relevance is very important for developing mainly proscriptive motives whereas prescriptive motives need both the relevance and the functionality. In other words, the relevance of the home is linked to readiness to perceive social norms and restrain oneself, and real objective features of convenient and comfortable home are connected with moral strivings, motives for doing something good. Overall, all connections were favorable, in accordance to our predictions.

Results from the regression analysis showed that functionality predicted moral motives very poorly, except for a positive connection between pragmatism and self-reliance. As for relevance, it contributed to the moral motives more heavily, in line with the outcomes presented above. Five out of six relevance features formed connections with the moral motives. No surprise that historicity predicted helping motive: people with high attachment to their homes are more likely to be altruistic. The opposite is also true: individuals who have not been living in their homes for a long time cannot use the home as a resource for their identities, and, as a result, have less mature personalities, in accordance with phenomenological understanding of interplay between a person and their home (Case, 1996; Manzo, 2003). Home detachment predicted social justice: this means that youth who perceive their homes as unreliable, filled with broken inconvenient equipment and furniture, and suffer from the domestic stress tend to restore social justice. In our opinion, this unexpected fact could discover some new aspects of social activism as well as of the genesis of justice motive, especially in youth.

We have also revealed some unexpected outcomes: self-presentation and management turned out to be anti-predictors of social order. Thus, people who can present themselves through their dwelling, and who can manage and change it, aren't ready to keep social order. Probably, they have enough order in their fitting and manageable homes, so that they aren't sensitive to the lack of order in the world. On the other hand, this unexpected fact might be caused by personalities of inhabitants: people striving for the self-presentation and managing their environments are often hysterical and narcissistic so that they like unpredictable world, free of rules and order. In any case, moral motives are predicted by the home environment and home representation, but we cannot state that the friendly home uniquely contributes to the rise of the moral motives level.

As like as it was shown earlier (Nartova-Bochaver S. et al., 2015), we also found that plasticity did not play an important role as a predictor of morality. Earlier we discovered that it was not related to parameters of well-being in youth (Nartova-Bochaver et al., 2016). This interesting fact may be better understood taking into consideration the age specificity of the sample: most students have not separated from their parents' family yet, and have to adapt to the shared household. They do not use plasticity of their flats but their parents mainly do; thus, they might compensate this dependence in the everyday life and domestic routine with the high orientation toward their inner, personal resources, such as self-reliance.

To sum up, we could note that prescriptive (activating) motives were more sensitive to the home environment's features than proscriptive ones. Thus, home rather stimulates inhabitants' morality than prevents them from sins.

We also investigated into whether the moral motives predicted the home representation or not. Interestingly, these impacts turned out to be stronger. Thus, social order, self-reliance, and helping/fairness contributed to all of the perceived functionality features—pragmatism, development, stability, and protection. To sum up, people who are ready to help others, are industrious, respect social order, are more likely to feel their homes as more functional, and, therefore, friendly to them.

Furthermore, we have revealed that moral motives heavily contributed to the perceived relevance of the home; almost all features of relevance were predicted by moral motives, and these connections were mostly easily interpretable. Only self-restraint contributed to home detachment, and historicity was not predicted by moral motives at all. This unexpected outcome could also be explained by the age peculiarity of the sample: in fact, self-restraint is not a youth's value, and the youth are more likely to act in a spontaneous way. Thus, young individuals who restrain themselves in their everyday lives might be predisposed to depressions and disappointments, and, as a result, perceive their homes through the lens of the domestic stress. Moreover, this result is in agreement with Maslow's ideas: people deprived in their basic needs are less likely to realize “higher,” including moral ones (Maslow et al., 1970).

One more result was found not to be easily interpreted: the helping motive impacted the perceived plasticity of the home negatively. It means that altruistic individuals who are ready for change their environments to the better feel their homes as stiff, and being in need of “enlivening” it due to their good activities. Let's once more take into account that helping is a prescriptive, activating motive.

Only historicity was not predicted by the moral motives. This fact seems to be quite interpretable: as respondents are youth, in according to their development task, they have to separate from the parental families, and to get their own familiar relationship and, probably, homes (Havighurst, 1972). The feeling of tight connectedness to the home and family (historicity) doesn't help in solving this task. Thus, it was not a surprise that historicity was not a significant feature and didn't form any connections in our study.

The results are very impressive. However they are in a full accordance with the Lens Model by Brunswik (1956) which stated that the person perceived the world (including all of its parts) through the lens of their attitudes toward the world. Morality is obviously one of the most important lenses or filters determining the people's representation of the world. In addition, they are in line with Morgan's Developmental Theory of Place Attachment (2010) demonstrating that positive attitudes toward the place and people who live there are inseparable from each other.

## Limitations of the study

Recent research was a preliminary entry into a topic and an effort to look at person-home interaction theme under a certain angle of view, namely, from the position of moral motives. We would outline the following prospects of the study, which would improve its convincingness. First, the future research steps are planned to be conducted on the more complicated manner, such as SEM. Secondly, the sample consisted mainly of female students. In the future research, it could be interesting to extend our sample due to male participants and adult and elderly people. Thirdly, our work does not deal with the real home but with home representations only; it damages the ecological validity of the study. In the future, we will try to take into account objective home characteristics. In the fourth place, we did not control a procedure of data collecting (at home or in class) which probably might influence the outcomes. However, at the same time, this allows a large number of intervening variables. These links could change if we invite more mature people with various experiences, family situations, and from different life conditions. This all opens the next prospects of investigation including possible interaction effects between age, gender, marital status, and administration of the survey.

## Conclusions

As expected, we revealed many connections between home representations and moral motives, and these connections were mainly positive. Simultaneously, moral motives were more pronounced in predicting the home environment in comparison with the impact of the home environment on moral motives. Generally, our hypothesis was confirmed.

On the one hand, results show that the home environment is actually connected with person's moral development, providing values, and skills of humane attitude to oneself, another person, and a group of people in general. This attitude seems to be embedded mainly in the relevance of the home. As for the functionality, only pragmatism had an impact on self-reliance motive. Thus, the friendlier and more welcoming the home is the more moral and altruistic the inhabitants are.

On the other hand, we revealed the backward connections as well which even seem to be more impressive. Hence, people with a high level of moral motives perceive their homes as friendly, convenient, safe places. This result is also easily interpretable: as the home is the most important part of the world, the “core” place in people's lives, it has been perceived through the lens of the inhabitants' worldview, including moral motives.

Despite some unexpected negative connections, overall, the study demonstrated mutual relationships between the home and its inhabitants, and most of these connections are reciprocal. Thus, we can conclude that people are in fact inseparable from their homes.

Our results seem to have applications in everyday life techniques and unguided self-therapy, especially, in the vicar territories, social institutions, dormitories, prisons, and so on. In addition, they are expected to be used in home design industry and social work, namely, in social expertise area. They seem to be useful in the family therapy because they discovered possible predictors of positive/negative attitudes toward home. Finally, they could become the basis for personal training programs.

## Author contributions

SN-B has made a substantial contribution to the conception and design of the study, she was participating in drafting the work, in final approval of the version to be published; to sum up, she fully agrees to be accountable for all aspects of the work in ensuring that questions related to the accuracy or integrity of any part of the work are appropriately investigated and resolved. VK has made a substantial contribution to the analysis and interpretation of data for the article, revising it critically for important intellectual content and approved finally the version to be published; and agrees to be accountable for all aspects of the work in ensuring that questions related to the accuracy or integrity of any part of the work are appropriately investigated and resolved.

### Conflict of interest statement

The authors declare that the research was conducted in the absence of any commercial or financial relationships that could be construed as a potential conflict of interest.
